# Supplementary material for: Empirical methods for controlling false positives and estimating confidence in ChIP-Seq peaks
Source: BMC Bioinformatics. 2008 Dec 5;9:523. doi: 10.1186/1471-2105-9-523 (PMC2628906; doi:10.1186/1471-2105-9-523)
Supplement: Additional File 1 — A variety of html documents from the USeq web site detailing the available applications, their best usage, output file type descriptions, command line menus, etc. [file 1471-2105-9-523-S1.zip › USeqUserGuides/JavaDoc.zip!/JavaDoc/gata/geneGlyphs/GenericGlyph.html]

GenericGlyph


|  |  |  |
| --- | --- | --- |
| **Overview** \| **Package** \| **Class** \| **Use** \| **Tree** \| **Deprecated** \| **Index** \| **Help** | |  |
| **PREV CLASS**   **NEXT CLASS** | **FRAMES**    **NO FRAMES**     **All Classes** |  |
| SUMMARY: NESTED \| FIELD \| CONSTR \| METHOD | DETAIL: FIELD \| CONSTR \| METHOD |  |


---


## gata.geneGlyphs Class GenericGlyph

```
java.lang.Object
  gata.geneGlyphs.GenericGlyph
```

---

``` public class GenericGlyph extends java.lang.Object ```

**Author:**
:   Nix
    Contains info related to drawing a GenericFeature, non standard gff annotation

---

**Constructor Summary**

`GenericGlyph(GenericFeature gf)`


| **Method Summary** | |
| --- | --- |
| `void` | `draw(float yCoord, float ntAtXZero, float trackThickness, float arrowHeight, float pixPerNt, java.util.ArrayList trackLabels, float letterHeight, float pixBtwLabelTrack, boolean trackLabelVis)` |
| `java.awt.geom.Rectangle2D.Double` | `getBoundingBox()` |
| `java.awt.Color` | `getColor()` |
| `GenericFeature` | `getGenericFeature()` |
| `java.awt.geom.GeneralPath` | `getPath()` |
| `double` | `getScore()` |
| `void` | `setColor(java.awt.Color color)` |
| `java.lang.String` | `toString()` |

**Methods inherited from class java.lang.Object**

`equals, getClass, hashCode, notify, notifyAll, wait, wait, wait`

**Constructor Detail**

### GenericGlyph

```
public GenericGlyph(GenericFeature gf)
```


**Method Detail**

### toString

```
public java.lang.String toString()
```

:   **Overrides:**: `toString` in class `java.lang.Object`

---


### draw

```
public void draw(float yCoord,
                 float ntAtXZero,
                 float trackThickness,
                 float arrowHeight,
                 float pixPerNt,
                 java.util.ArrayList trackLabels,
                 float letterHeight,
                 float pixBtwLabelTrack,
                 boolean trackLabelVis)
```

---


### getPath

```
public java.awt.geom.GeneralPath getPath()
```

---


### getBoundingBox

```
public java.awt.geom.Rectangle2D.Double getBoundingBox()
```

---


### getColor

```
public java.awt.Color getColor()
```

---


### getScore

```
public double getScore()
```

---


### setColor

```
public void setColor(java.awt.Color color)
```

---


### getGenericFeature

```
public GenericFeature getGenericFeature()
```


---


|  |  |  |
| --- | --- | --- |
| **Overview** \| **Package** \| **Class** \| **Use** \| **Tree** \| **Deprecated** \| **Index** \| **Help** | |  |
| **PREV CLASS**   **NEXT CLASS** | **FRAMES**    **NO FRAMES**     **All Classes** |  |
| SUMMARY: NESTED \| FIELD \| CONSTR \| METHOD | DETAIL: FIELD \| CONSTR \| METHOD |  |


---
